# The Complex Functions of the NME Family—A Matter of Location and Molecular Activity

**DOI:** 10.3390/ijms222313083

**Published:** 2021-12-03

**Authors:** Uwe Schlattner

**Affiliations:** 1 University Grenoble Alpes and Inserm U1055, Laboratory of Fundamental and Applied Bioenergetics (LBFA), 38058 Grenoble, France; uwe.schlattner@univ-grenoble-alpes.fr; 2 Institut Universitaire de France (IUF), 75231 Paris, France

The family of NME proteins represents a quite complex group of multifunctional enzymes. This is also reflected by their nomenclature, since members are either called nucleoside diphosphate kinases (NDPK) based on the enzymatic activity of the first described members, or else NME (non-metastatic protein) or NM23 (non-metastatic clone 23) based on the metastasis suppressor function of some family members. This editorial will refer to the family and its members exclusively as NME. 

First described almost 70 years ago, and present from bacteria to man, there are now 10 different NMEs known in vertebrates [1]. They display a large range of physiological and pathological functions at the cellular and organ levels, including bioenergetics, cytoskeleton and membrane dynamics, cell signaling, DNA repair, metastasis, maintenance of vascular and cardiac function, and development in general. Many NME proteins are multifunctional, i.e., they present more than one and often unrelated molecular activities (for earlier reviews, see [2] and the related article collection [3]). This obviously raises many questions about the biological meaning of this NME diversity and the precise molecular mechanisms behind the various functions. These and other pertinent and timely questions have been addressed at the 11th International Congress on the NME/NDPK/NME/AWD protein family (NME2019) that was held in October 2019 in Talloires, French Alpes. Selected contributions, including original research and review articles, have been published in a Special Issue of the *International Journal of Molecular Sciences* (IJMS) in 2020 and 2021 [4]. This editorial puts these contributions (identified here by the first author and affiliations) into the context of the above stated questions and of some further most recent advances in the field. It emerges that cellular localization and interaction with specific proteins, membrane lipids or DNA, together with few basic molecular activities are pivotal for NME biology.

Basically, the 10 NME isoforms can be subdivided into two groups [1]. The quite conserved and ubiquitously expressed members NME1-4 (group I) form hexamers and have NDP kinase activity. They have been largely known for decades, and comprise the best studied and most abundant NME1 and NME2. The more divergent members NME5-10 (group II) probably lack both hexameric structure and NDP kinase activity. Some contain NDPK domain duplications and further domains with mostly unknown function. Only NME6 is ubiquitously expressed, most others were reported in association with ciliary and flagellar structures. Our current knowledge on structure, folding and stability of NMEs, based on available X-ray structures and biophysical analysis is reviewed in much detail by Georgescauld et al. (Northeastern University, Boston, MA, USA, and Univ. Bordeaux, France [5]). While clearly there is a good understanding of the group I NME structures and their classical functioning as NDP kinases, essential for generating the different cellular (d)NTPs, little is known about the structural basis of other described functions of NME proteins and their interactions with specific partners.

**Figure 1:**
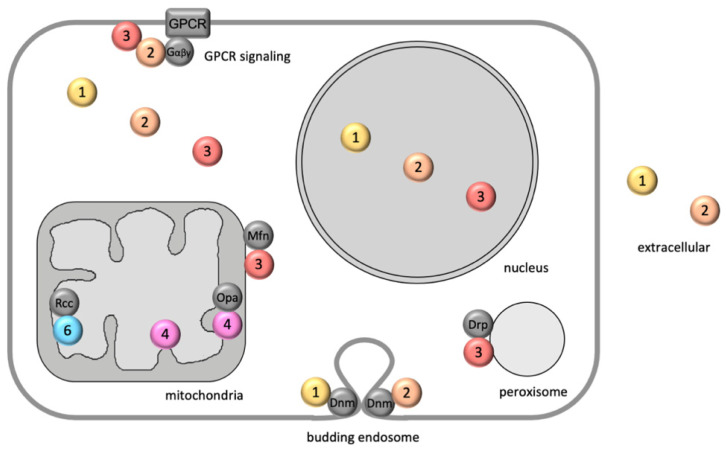
Localization and interaction partners of ubiquitously expressed NME family members in a schematic eukaryotic cell. NME proteins 1, 2, 3, 4 and 6 are shown as colored circles. Note that NME1-4 form hexamers, with cytosolic NME1-3 likely occurring mainly as heterohexamers [56]. Some of the known NME-interacting proteins are shown as dark grey circles. Members of the dynamin superfamily: Mfn, mitofusins 1 and 2 (Mfn1/2), Opa, optic atrophy protein 1 (Opa1), Drp, dynamin-related protein 1 (Drp1), Dnm, dynamin 2 (DNM2). Other proteins: GPCR, G-protein-coupled receptor; Gαβγ, heterotrimeric G protein; Rcc, RCC1-like G exchanging factor-like protein (RCC1L or WBSCR16). For further details see text.

Cellular localization is emerging as a major factor defining NME function (Figure 1). It is mainly driven by specific binding to proteins, phospholipids or DNA, localizing NMEs, e.g., to membranes or chromatin. In a few cases, there is a signal sequence, such as the N-terminal mitochondrial targeting sequence of NME4. Out of the ubiquitously expressed NMEs, NME1, 2 and 3 are found in the cytosol, but they also distribute into the nucleus. How this works exactly is not known, since these NMEs lack a nuclear localization sequence. However, the process seems to be regulated. Radić et al. (RBI, Zagreb, Croatia [6]) describe how radiation-induced damage localizes homo- and heterohexameric NME1/2 to the nucleus. NME3 has a N-terminal hydrophobic peptide that allows its anchoring to biological membranes, with a possible involvement of phosphatidic acid [7]. Indeed, NME3 can bind to the cytosolic face of the plasma membrane [8], the outer mitochondrial membrane [9] and, as shown by Honsho et al. (Kyushi University, Fukuoka, Japan [10]), the peroxisomal membrane. Mitochondria emerge as a key compartment of NME diversity. While NME3 attaches to the mitochondrial surface, NME4 is imported into these organelles and localizes mainly to the intermembrane space, but is also found in the matrix [11]. In both compartments, NME4 is mainly bound to the inner mitochondrial membrane, interacting with cardiolipin, the mitochondrial signature phospholipid [11]. Most recent data reveal that NME6, the only ubiquitously expressed group II NME, also localizes to the mitochondrial inner membrane, mainly facing the matrix [12]. In contrast to NME4, NME6 is monomeric and NDP kinase inactive. Finally, cytosolic NME1 and NME2 are enriched in particular in the extracellular space of many tumors, but whether this involves a regulated export process is still a matter of debate (for recent reviews see [13,14]). At many of these cellular locations, NME proteins interact structurally and/or functionally with specific protein partners to fulfill their function (see Figure 1). Some unifying molecular mechanisms emerge, which include in addition to classical phosphotransfer to NDPs (NDP kinase) also phosphotransfer to protein histidine residues (protein kinase), interaction with DNA nucleotides with roles in DNA synthesis, proofreading, repair and transcription, interaction with phospholipids for membrane anchoring and phospholipid transfer, and eventually even further mechanisms.

The canonical activity of group I NMEs is phosphotransfer from NTPs (physiologically mainly ATP) to NDPs (physiologically mainly GDP), which involves transient phosphorylation of the NME active site at the essential His118. This NDP kinase function is certainly important for global cellular nucleotide homeostasis. Since NTPs other than ATP are much less abundant than ATP, efficient and constant replenishment of these cellular NTP pool is essential, and group I NMEs are the major enzymes in this pathway. However, NDP kinase activity is also involved in the direct fueling of GTP-dependent cellular processes [15]. Such fueling occurs locally, by NMEs directly interacting with GTP-binding proteins and GTPases. An emerging prototype case is GTP-fueling of the dynamin superfamily, a group of related GTPases involved in different forms of membrane dynamics. At the plasma membrane, NME1/2 interact with dynamin for maximal efficiency of endocytosis [16]. This concept has been extended in recent years to other dynamin-related GTPases localized in mitochondria and peroxisomes [9,16,17]. In mitochondria, interaction of NME4 with OPA1 at the inner membrane [11], and of NME3 with Mfn at the outer membrane [9] are required for fusion of these organelles into filaments and networks. In case of NME3, a homozygous mutation in the coding gene can lead to a neurodegenerative disorder [9]. Interestingly, also kinase-dead NME3 was efficient for fusion, suggesting that it may recruit active NME1 or NME2 into heterooligomers at the mitochondrial surface. In line with these findings, Chen et al. (Taiwan National University, Taipei, Taiwan [18]) now report that NME3 knock-down leads to mitochondrial fragmentation, which finally also affects mtDNA via mitochondria-generated ROS. Even more so, Imoto et al. (Johns Hopkins University, Baltimore, MD, USA, and Kyushu University, Fukuoka, Japan [19]) review the role of yet another dynamin-related GTPase, DRP1, for fission of both mitochondria and peroxisomes. By using the unicellular red algae *C. merolae* as a model system, the authors evidence a role for the NME3-homolog DYNAMO for fueling Drp1 [17]. Honsho et al. (Kyushu University, Fukuoka, Japan [10]) further provide evidence that mammalian NME3 can indeed function analogous to DYNAMO in channeling GTP to DRP1 for peroxisome fission. If such GTP fueling of DRP1 also occurs for mitochondrial fission in mammals remains to be shown. Thus, across different membranes in different compartments, NME proteins can locally fuel associated dynamin-related GTPases.

Phosphohistidine is a crucial post-translational modification ubiquitous to both prokaryotes and eukaryotes [20]. Class I NMEs, in particular NME1/2, can transfer phosphate from their active site His not only to NDPs, but also to protein histidine residues, thus acting as protein kinases. Adam et al. (Salk Institute, La Jolla, CA, USA [21]) introduce this long-time underestimated and largely understudied part of cell signaling. They then evaluate the potential role of class I NMEs, in particular NME1 and NME2, as protein histidine kinases. Using the recently generated anti-P-histidine antibodies, Adam et al. (Salk Institute and UC San Diego, La Jolla, CA, USA [22]) further analyze histidine phosphorylation in neuroblastoma, where the genes for NME1 and NME2 are located in an amplified chromosome region.

Nuclear NME1 and NME2 interact with DNA in a non-sequence-specific manner, mainly directed to single-stranded regions. This has been linked to local NTP synthesis for DNA polymerase activity, but also to non-canonical NME activities such as 3′-5′ exonuclease (NME1), required for DNA proofreading and repair, or different transcription factor/co-regulator activities (NME1/2) (for a recent review see [23]). NME1 and NME3 are also recruited to sites of DNA damage where they play a role in the repair of DNA single- and double strand breaks. Puts et al. (University of Maryland, Baltimore, MD, USA [24]) describe the role of NME1 in the repair of DNA double-strand breaks, in particular the choice of the repair pathway. Nuclear NMEs can also interact with telomers, and Sharma et al. (CSIR, New Delhi, India [25]) review the role of telomer-localized NMEs for regulating telomer-related factors. There may be even further, rather unexpected molecular interactions of NME proteins with nucleotide-containing molecular structures. For example, NME1 binding to the key metabolite Coenzyme A (CoA) was reported, which is competitive in respect to nucleotides, and can lead to an inhibitory covalent CoA-modification of NME1 [26]. Most recently, mitochondrial NME6 was mainly localized in complexes with the regulator of chromatin condensation 1-like protein (RCC1L, also called WBSCR16) [12] that is involved in mitoribosome biogenesis and thus mitochondrial translation. RCC1L is part of a large GTP-binding protein family, but it is unknown whether it can function as a GDP/GTP exchange factor, and which would be the role of the kinase-inactive NME6 within a likely multi-protein regulatory complex. All these nucleotide-related NME activities certainly deserve more detailed analysis, in particular for their structural basis and contributions in cancer and metastasis-suppression.

Further NME activities depend on interactions different from those with nucleotides or protein histidines. Indeed, at least all class I NMEs have a certain affinity to membrane phospholipids. While NME3 may be recruited to the mitochondrial outer membrane via its hydrophobic anchor and phosphatidic acid [7], NME4 binds to cardiolipin, a mitochondria-specific phospholipid synthesized at the mitochondrial inner membrane [11]. This binding is not only required for its OPA1 interaction and GTP fueling [16,27], it also facilitates the intermembrane transfer of cardiolipin to the mitochondrial surface as part of a novel lipid-signaling pathway [28]. Such cardiolipin externalization can trigger mitophagy or apoptosis, depending on additional factors [27,29]. Surface-exposed cardiolipin has been also involved in the opening the mitochondrial permeability transition pore and the formation of the Nlrp3 inflammasome, a multiprotein complex that generates proinflammatory signals [30]. A recent genome-wide screen for inflammasome regulators in macrophages identified the requirement of NME4 not only for mitochondrial DNA synthesis, but also for cardiolipin externalization and inflammasome assembly [31]. Collectively, these data suggest that NME4-supported cardiolipin externalization is crucial for different signaling pathways. Interestingly, the screen for inflammasome regulators identified also the other two mitochondrial NMEs, NME3 and NME6, with hitherto unknown roles in inflammasome activation. 

Out of the many NME functions at the cellular and organ level, inhibition of metastatic dissemination of cancer cells is certainly one of the most important. NME1 has been the first metastasis suppressor described, and this has also been proposed for NME2. By genetically manipulating NME1 and NME2 in different cancer cells, Huna et al. (University of Lyon, and Sorbonne, Paris, France [32]) provide now evidence that only NME1, but not NME2 can inhibit early stages of metastasis. Most recently, NME4 was also identified as the first mitochondrial metastasis suppressor [33]. However, the mechanistic basis of the anti-metastatic role of NMEs is not yet fully understood and likely comprises multiple molecular activities [34]. In case of NME1/2, these include the management of cell surface receptors and metalloproteases by NME-controlled, dynamin-dependent endocytosis [16,34,35]. This control likely occurs via GTP fueling [16], but other activities seem to contribute, including support of dynamin oligomerization [34]. Work on the *Drosphila* NME1/2 homologue Awd showed how altered receptor endocytosis can affect downstream signaling affecting cell motility and migration, and more generally development, for example via Notch signaling. Serafini et al. (University of Bologna, Italy [36]) show that loss or downregulation of Awd results in a dose-dependent loss of Notch signaling and affects *Drosphila* Wingless signaling. In an alternative anti-metastatic signaling pathway, NME1 interacts with p110α, a catalytic subunit of phosphoinositide 3-kinase (PI3K), to inhibit downstream PI3K-Akt signaling by so far unclear mechanisms [37]. In case of metastasis suppression by NME4, a primary mechanism is likely the maintenance of a fused mitochondrial network via NME4-controlled, Opa1-dependent mitochondrial fusion [33], analogous to the NME1/2-controlled dynamin-dependent endocytosis. Loss of NME4 induces fragmentation of the mitochondrial network, which together with altered mitochondria-nuclear retrograde signaling and gene expression favors metastasis. Increasing the levels of anti-metastatic NMEs could be a therapeutic option. Felix et al. (Universities of Ulm and Heidelberg, Germany [38]) present proof-of-principle for using the toxins transport component PA63 of *Bacillus anthracis* for delivery of recombinant human NME1 into cultured human cancer cells. Another approach may be the stabilization of the NME1 hexameric structure [39].

Interestingly, extracellular NME1/2 seem to have effects contrary to their cytosolic location. They rather promote cancer and metastasis, and inhibit differentiation as, e.g., in case of stem cells or hematopoietic progenitor cells [13,14]. At least these latter effects may be independent of NDP kinase activity, and could involve unknown receptor-based signaling. In breast cancer, however, there is evidence that NME2 associated with extracellular vesicles can perturb nucleotide-dependent purinergic signaling, likely based on its NDP kinase activity [40]. Additionally, some pro- and eukaryotic pathogens secrete NMEs to escape host protective responses [41], among others by interfering with the extracellular purinergic signaling of the host cell. The precise mechanisms of extracellular NMEs certainly require further investigation.

NME proteins also play important roles for the cardiovascular system. Cytosolic, membrane-anchored NME2/NME3 heterotrimers favor GTP/GDP exchange at the alpha subunit of heterotrimeric G-proteins, necessary for signaling downstream of G-protein-coupled receptors (GPCR) [8,42]. This occurs not by classical GTP fueling of the G-proteins, but rather via rephosphorylation of G-protein-bound GDP by a so-called phosphohistidine relay from a phosphorylated NME, which would thus work as protein histidine kinase [43]. For other cardiovascular functions of cytosolic NMEs, the molecular basis is less clear. NME2 favors surface accumulation of caveolins and thus the formation of caveolae required for the functionality of many GPCRs [43]. It also contributes to the endothelial barrier function via an NDP kinase-independent modulation of protein O-GlcNAcylation [44] and vascular growth factor angiotensin 2 [45], for which Chatterjee et al. (University of Heidelberg, Germany [46]) have now identified a possible mechanism.

Some progress has also been made for other group II NMEs. Mutation in NME5 was repeatedly observed as a cause for primary ciliary dyskinesia in animals and human [47,48,49]. Semi-lethal primary ciliary dyskinesia was also reported for an NME7 knock-down in rats [50], suggesting similar functions of NME5 and NME7 in ciliogenesis and control of ciliary transport in mammals. Further studies on rat models of metabolic syndrome or heterozygous NME7 knock-out suggest a link between NME7 and glucose intolerance or adiposity [51,52]. NME7 in the red algae *Chondrus crispus*, an early eukaryote, differs from its human homologue in its oligomeric structure and possibly in function [53]. Finally, a most recent study identifies NME7 as an activator of Wnt/β-catenin signaling in hepatocellular carcinoma, based on a hitherto unknown serine protein kinase activity of NME7 [54].

Taken together, a much more precise picture of the NME family emerged during recent years, in particular concerning cellular location, interaction partners and function of several members. However, many aspects of NME biology remain unsolved. This concerns in particular the less studied NME family members, first of all the group II NMEs. For group I NMEs, major questions concern the importance of the non-NDP kinase functions. For example, what is the role of NME protein histidine kinase activity in the cell? Is phospholipid binding and transfer limited to NME4? Additionally, are there further, still unknown activities, that may explain NME functions that are apparently independent of phosphotransfer? Possibly, the most generalizable function of NME proteins could be the scaffolding of different interaction partners as proposed earlier [55]. So much of what has to be learned about NME proteins is still ahead of us.
